# Synthesis and Vasorelaxant Effect of 9-aryl-1,8-acridinediones asPotassium Channel Openers in Isolated Rat Aorta

**Published:** 2012

**Authors:** Mohsen Imenshahidi, Farzin Hadizadeh, Asieh Firoozeh-Moghadam, Mahmoud Seifi, Atefeh Shirinbak, Mohammad Bagher Gharedaghi

**Affiliations:** a *Pharmacy Faculty, Mashhad University of Medical Sciences, Mashhad, Iran.*; b *Biotechnology Research Center, Mashhad University of Medical Sciences, Mashhad, Iran*.

**Keywords:** Potassium channels, Isolated, Vasorelaxant, ATP-sensitive potassium

## Abstract

ATP-sensitive potassium (K_ATP_) channel openers have a relaxation effect due to the lower cellular membrane potential and inhibit calcium influx. There has been considerable interest in exploring K_ATP_ channel openers in the treatment of various diseases such as cardiovascular, cerebrovascular, and urinary system disease and premature labor. The purpose of this study was to synthesize 3,3,6,6-tetramethy l-9-aryl-octahydro-1,8-acridindiones and investigate their effects on vascular potassium channels and mechanism of induced relaxations on phenylephrine-induced contractile responses in isolated rings of rat aortic smooth muscle.

In this study, four new derivatives of 3,3,6,6-tetramethy l-9-aryl-octahydro-1,8-acridindione [2a-d] were synthesized by the reaction of 5, 5-dimethyl-1,3-cyclohexanedione with an aromatic aldehyde, 2-alkylthio-1-(4-fluorobenzyl)-5-formylimidazole or 3-substituted benzaldehyde, in the presence of ammonia in methanol. Their effects on vascular potassium channels and mechanism of induced relaxations on phenylephrine-induced contractile responses in isolated rat aorta were investigated. Minoxidil was used as a standard potassium channel opener and Glibenclamide was used as a standard potassium channel blocker. The effects of compounds on KCl-induced contractile response which is an indicator of ca-channel blocking activity was also investigated and compared to that of nifedipine as a standard calcium channel blocker.

Compounds 3a-d and Minoxidil relaxed the contractions exerted by using phenylephrine with the potency order as follows: Minoxidil > 3c > 3d > 3a > 3b. This effect was sensitive to the potassium channel blocker Glibenclamide. It can be concluded that these compounds act via ATP-sensitive potassium (K_ATP_) channels. Selectivity index (SI) for these compounds and Minoxidil also shows that these compounds are selective to ATP-sensitive potassium (K_ATP_) channels and the selectivity of compounds 3a-d is less than Minoxidil.

## Introduction

It is well known that ion channels are very important for cell function and responsible for physiological effects. Potassium channels regulate some functions in both excitable and non-excitable cells. Potassium channel opening is a physiological mechanism by which the excitable cells exploit to maintain or restore their resting state (1).

K_ATP_ channel (ATP-sensitive K channel) openers are a structurally diverse group of drugs with a broad spectrum of potential therapeutic usages. These drugs interact with K_ATP_ channels in numerous tissues and increase their activity, thereby hyperpolarize the plasma membrane and reduce electrical excitability (1). Minoxidil and diazoxide are two examples of vasorelaxant drugs that act via opening ATP-sensitive K channel. 

Many mammalian aorta cells have two distinct ATP-sensitive potassium (K_ATP_) channels. The classic one is in the surface membrane (sK_ATP_) and the other one is in the mitochondrial inner membrane (mitoK_ATP_). Cardiac (mitoK_ATP_) channels play a vital role in ischemic preconditioning and thus represent interesting drug targets (2-6). They are also important in the control of vascular tone and blood pressure. 

The dihydropyridine system is usually associated with calcium L-channel blockade and activation. This class of compounds have been the subject of many structure-activity relationship (SAR) studies (7-10) and the recent developments in the chemistry of DHPs has been reviewed (11). The potassium channel in particular has several general features analogous to the calcium channel (12). 1,4-Dihydropyridine (DHP) derivatives and their bicycle (quinoline) and tricyclo (acridine) analogs are a well known group of calcium channel blockers. They are used in the clinic as vasodilator and antihypertensive. 1, 4-Dihydropyridine derivatives have also potassium channel opener activities (13-15). 

For example, niguldipine, which is a 1,4- DHP derivative, has increased K^+^ flux in isolated vascular smooth muscle by opening Ca^2 +^- activated potassium channels(1, 15). 

The purpose of this study was to synthesize 3,3,6,6-tetramethyl-9-aryl-octahydro-1,8- acridindiones and investigate their effects on vascular potassium channels and mechanism of induced relaxations on phenylephrine-induced contractile responses in isolated rings of the rat’s aortic smooth muscle. 

## Experimental

*Chemistry*

Melting points were determined on Electrothermal Capillary apparatus and are uncorrected. The IR spectra were obtained using a Perkin-Elmer Model 1000. One H nuclear magnetic resonance (NMR) was obtained on Bruker Ac-80 spectrophotometer and chemical shifts () are in ppm relative to internal tetramethylsilane. C, H, N analyses were within ± 0.4% of theoretical values. Title compounds 3a-d were sensitive to light and all chemical procedures involving these were shielded from light whatever present. Compounds 2a-d was prepared as described previously (16). 

*3,3,6,6-Tetramethyl-9-[1-(4-fluorobenzyl)-2-(methylthio)-5-imidazolyl]-2,3,4,5,6,7,9,10- octahydro-1,8-acridinedione [3a]*

A mixture of ammonium acetate (0.32 g, 0.41 mmol), 2a (1 g, 0.41 mmol) and 5, 5-dimethyl- 1,3-cyclohexanedione (1.18 g, 0.84 mmol) in methanol (15 mL) was protected from light and refluxed overnight. Then the residue was poured in ice-water. The obtained precipitate was filtered to give 0.4 g of 3a , m.p. 111.1°C, yield 88.7%; IR (KBr): 1630 cm^-1^ (C=O); 1H nmr (DMSO-d_6_): δ 7.83-6.80 (m, 6H, arom, NH, H_4_-imidazole), 6.20 (s, H_4_-DHP), 5.00 (s, 2H, CH_2_N), 2.80-1.80 (m, 11H, CH_2_, CH_3_S), 1.00 ppm (s, 12H, CH_3_). 

Anal. Calcd. for C_28_H_32_FN_3_O_2_S: C, 68.13; H, 6.53; N, 8.51.Found: C, 68.09; H, 6.63; N, 8.48. 

*3,3,6,6-Tetramethyl-9-[1-(4-fluorobenzyl)-2-(ethylthio)-5-imidazolyl]-2,3,4,5,6,7,9,10- octahydro-1,8-acridinedione [3b]*

This compound was prepared from 2b similar to 3a, m.p. 95.86 °C , yield 94.2% ; IR (KBr): 1632cm^-1^ (C=O); 1H nmr (DMSO-d_6_): δ 7.41- 6.40 (m, 6H, arom, NH, H_4_-imidazole), 6.00(s, H_4_-DHP), 4.90(s, 2H, CH_2_N), 2.90-1.60 (m, 10H, CH_2_, CH_2_S), 1 ppm (m, 15H, CH_2_, CH_3_). 

Anal. Calcd. for C_29_H_34_FN_3_O_2_S: C, 68.61; H, 6.75; N, 8.28. Found: C, 68.53; H, 6.84; N, 8.39.

*3,3,6,6-Tetramethyl-9-(3-cyanophenyl)-2,3,4,5,6,7,9,10-octahydro-1,8-acridinedione [3c]*

This compound was prepared from 2c similar to 3a, m.p 182.3°C, yield 96.5%; IR (KBr): 1628 cm^-1^ (C=O); 1H nmr (DMSO-d_6_): δ 7.70-6.80 (m, 4H, arom), 5.75 (s, H_4_-DHP), 2.60-1.50 (m, 8H, CH_2_), 1.20-0.30 ppm (m, 12H, CH_3_).

Anal. Calcd. C_23_H_26_N_2_O_4_: C, 70.03; H, 6.64; N, 7.10. Found: C, 70.13; H, 6.53; N, 7.08.

*Pharmacology*

Phenylephrine hydrochloride, Glibenclamide, nifedipine and Minoxidil were supplied by Sigma. Glibenclamide, nifedipine and compounds (3a-d) were dissolved in dimethyl sulfoxide (DMSO). Phenylephrine and KCl were dissolved in distilled water. DMSO in organ baths did not affect smooth muscle relaxations induced by compounds. All drug solutions were prepared daily.

In this study we used male Wistar rats (Razi Institutes, Mashhad, Iran), weighing 250-300 g. All animal procedures were approved by the ethical committee of Mashhad University of Medical Sciences. Animals were anesthetized with intraperitoneal injection of sodium thiopental (80 mg/Kg) and their thoracic aorta was removed, cleaned of adhering fat and cut into rings of 3-4 mm long. All rings were mounted under 2 g resting tension on stainless steel hooks in 20 mL organ baths. These organ chambers were filled with Krebs-Henseleit solution (KHS), with a composition (in mM) of: NaCl 118, KCl 4.7, MgSO_4 _2 H_2_O 1.2 KH_2_PO_4_, 2 H_2_O 1.2, NaHCO_3_ 25, CaCl_2_ 2.5 and glucose 11.1., aerated with a mixture of 95% O2/5% CO_2_ and kept in 37°C. Tension was measured isometrically through a force transducer (Grass FTO_3_C) and recorded continuously using a transducer amplifier (Janssen Scientific Instruments) and a pen recorder. After mounting, the preparations were allowed to equilibrate for 1 h.

Aortic rings were pre-contracted with 1 μM phenylephrine or 80 mM KCl and concentration-response curve for compounds (3a-d). Nifedipine and Minoxidil were obtained through cumulative addition of these drugs to the bath solution. The relaxant effects of the compounds were expressed as the percentage of precontraction using phenylephrine or KCl. To evaluate the effects of the compounds, PD2 values were calculated.

In addition, these experiments were performed in the presence of Glibenclamide (ATP-sensitive potassium channel inhibitor) (3×10-6M) (17).

Results were expressed as the mean ± SEM and were analyzed by one-way analysis of variance (ANOVA) followed by a Tukey-Kramer multiple comparison test. A p-value < 0.05 was considered to be significant.

## Results and Discussion

*Chemistry*

**Figure 1:**
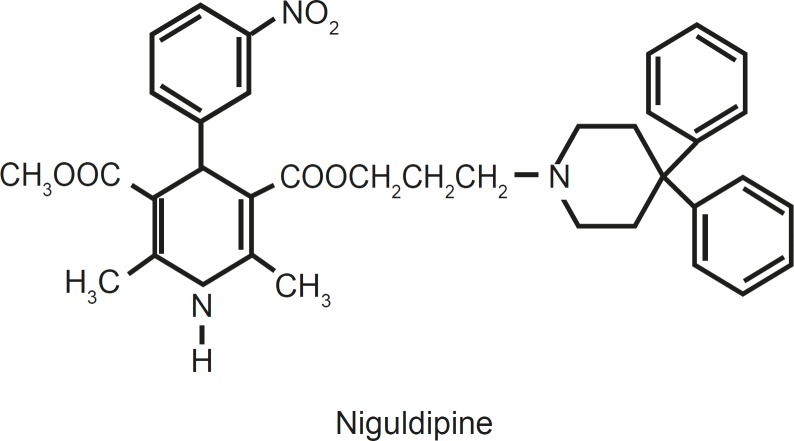
Niguldipine

Acridine derivatives (3a-d) have been prepared by the reaction of 5,5-dimethyl-1,3-cyclohexanedione (1) with aromatic aldehydes (2a-d) in the presence of ammonia in methanol (12) (Figure 1).

**Figure 2:**
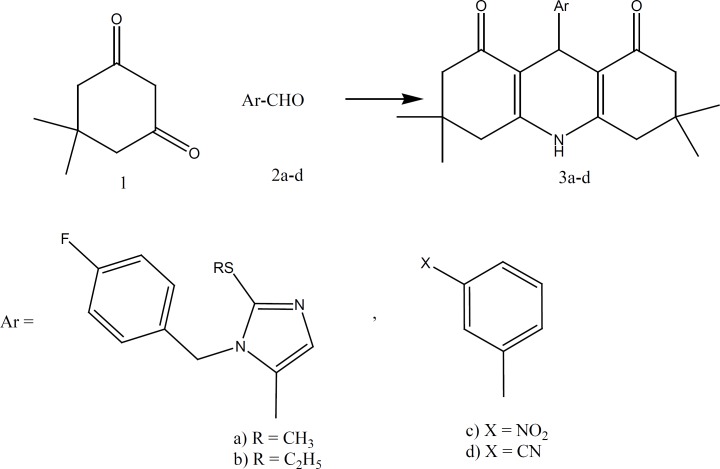
Synthesis of compounds 3a-d

The purity of the compounds was confirmed through TLC. The structure of the compounds was elucidated by IR, 1H-NMR and elemental analyses. All spectral data are in accordance with assigned structures. In IR spectra, N-H and C-O stretching bands were observed at spectra expected values. In the 1H-NMR spectra, methyl protons were seen at 0.90-1.00 ppm as separated singlets. Aromatic, methylene, methine and NH protons were seen at expected values.

*Pharmacology*

The relaxant effect of the test compounds 3a-d on isolated rings of rat aortic smooth muscle pre-contracted with KCl was given in Table 1. Comparison of pD2 value [the negative logarithm of the concentration for the half-maximal response (EC_50_)] of compounds with nifedipine shows that their potency in the case of KCl contraction is less than nifedipine. Regarding the mechanism of contraction induced through KCl, which activates L-type calcium channels of smooth muscles (18). It seems that the activity of these compounds on L-type Calcium channels is weaker than nifedipine. The order of pD2 for these compounds in relaxation responses in KCl-contracted aortic rings is: nifedipine > 3c > 3d > 3b > 3a.

**Table 1:** Relaxant effects of Minoxidil and compounds 3a-d on KCl or Phenylephrine-induced contraction

**Compound**	**Contractile Stimulus**	pD2*	**p-value (vs Phenylephrine group)**
**3a**	KCl Phenylephrine Phe** + Glibenclamide	3.964 4.675 3.788	*** ***
**3b**	KCl Phenylephrine Phe** + Glibenclamide	4.038 4.650 3.928	*** ***
**3c**	KCl Phenylephrine Phe** + Glibenclamide	4.047 5.175 3.988	*** ***
**3d**	KCl Phenylephrine Phe** + Glibenclamide	4.042 5.173 4.114	*** ***
**Minoxidil **	KCl Phenylephrine Phe** + Glibenclamide	---- 6.155 4.729	***

*Relaxation is expressed as pD2 of precontraction induced by KCl (80 mM) or Phenylephrine (1 μM) (in the absence or presence of Glibenclamide (10 μM)); pD2 value represent the mean value ± SE for 6 experiments; ** Phe: Phenylephrine;*** p ≤ 0.001 when compared with Phenylephrine.

The relaxant effect of the test compounds 3a-d and Minoxidil on isolated rings of rat aortic smooth muscle pre-contracted with phenylephrine were given in Table 1. Comparison of pD2 value of compounds in phenylephrine and KCl pre-contracted preparations shows that their potency in case of phenylephrine contraction is higher than KCl contraction. Therefore, it seems that the activity of these compounds on L-type calcium channels is weaker in comparison to their activity on potassium channel. The order of pD2 for these compounds in relaxation responses in phenylephrine-contracted aortic rings is: Minoxidil > 3c > 3d > 3a > 3b.

In the presence of Glibenclamide, pD2 values of compounds were decreased.

It can be concluded that the relaxation by using these compounds is sensitive to ATP-sensitive potassium channels (K_ATP_ channels) (17). In comparing compounds 3a-d to Minoxidil, the results of our study show that the potencies of all tested compounds are less than Minoxidil.

The calculating of SI index for these compounds by the following formula also shows that these compound are selective to ATP-sensitive potassium (K_ATP_) channels and this selectivity is less than Minoxidil.

SI (for potassium channels)=EC50 in the presence of GlibenclamideEC50 in the absence of Glibenclamide

It can be concluded from these data that compounds 3a-d are K_ATP_ channel (ATP-sensitive K channel) openers but their selectivity are less than Minoxidil as a standard K_ATP_ channel opener.
